# O.1.2-2 Delivering enjoyable group-based physical activity programmes to older adults: a qualitative exploration of the views of exercise instructors

**DOI:** 10.1093/eurpub/ckad133.089

**Published:** 2023-09-11

**Authors:** Rachel M Creighton, Nicole E Blackburn, Kyle F Paradis, Mark A Tully

**Affiliations:** Ulster University, UK; Creighton-R3@ulster.ac.uk; Ulster University, UK; Creighton-R3@ulster.ac.uk; Ulster University, UK; Creighton-R3@ulster.ac.uk; Ulster University, UK; Creighton-R3@ulster.ac.uk

## Abstract

**Purpose:**

Enjoyable group-based physical activity programmes provide an engaging opportunity for older adults to experience healthy ageing promotion. To understand the programme delivery context, we explored how exercise instructors seek to make their existing sessions enjoyable.

**Methods:**

A semi-structured interview guide was used to qualitatively investigate how exercise instructors create the conditions to promote an enjoyable experience. Exercise instructors (*n* = 9) who were experienced in delivering group-based exercise programmes to community-dwelling older adults were interviewed. The mean interview length was 50 minutes.

**Results:**

Reflexive thematic analysis generated three main themes: 1) Identifying individual expectations and motives for engagement. Exercise instructors recognised that safety was a foundational aspect of the exercise programme, so that older adults could enjoy exercising confidently. 2) Adopting a tailored approach to meet the needs of older adults. Exercise instructors ensured that the exercises were inclusive of all abilities so that older adults could enjoy exercising collectively. 3) Encouraging collective participation through the development of social support networks. Exercise instructors created a relaxed social atmosphere, facilitating social support, so that older adults could enjoy being part of the group and experience belonging.

**Conclusions:**

In summary, exercise instructors reported that tailoring sessions to meet the identified needs of older adults and encouraging the group to foster social support can create the conditions to promote an enjoyable experience. The findings can inform the promotion of enjoyment and engagement into existing programs. Findings can be used by exercise instructors, policy makers, researchers, and civil society organisations to ensure that opportunities for healthy ageing are sustainable. Longer-term group-based physical activity opportunities are required to ensure that older adults can experience belonging in a group they enjoy being active in, and which encourages the development of external social networks they may not otherwise have. For the purpose of the presentation, the primary focus is on how exercise instructors can incorporate the social elements of enjoyment in group-based physical activity programmes for older adults, across the dynamic progression of group identity.

**Support/Funding Source:**

This study was funded by a Postgraduate Studentship from the Department for the Economy (DfE) in Northern Ireland.

